# Lessons from Recent Advances in Ischemic Stroke Management and Targeting Kv2.1 for Neuroprotection

**DOI:** 10.3390/ijms21176107

**Published:** 2020-08-25

**Authors:** Chung-Yang Yeh, Anthony J. Schulien, Bradley J. Molyneaux, Elias Aizenman

**Affiliations:** 1 Department of Neurobiology, University of Pittsburgh School of Medicine, Pittsburgh, PA 15213, USA; chy64@pitt.edu (C.-Y.Y.); Schulien.Anthony@medstudent.pitt.edu (A.J.S.); 2 Pittsburgh Institute for Neurodegenerative Diseases, University of Pittsburgh School of Medicine, Pittsburgh, PA 15213, USA; moly@pitt.edu; 3 UPMC Stroke Institute, University of Pittsburgh Medical Center, Pittsburgh, PA 15213, USA; 4 Department of Neurology, University of Pittsburgh School of Medicine, Pittsburgh, PA 15213, USA

**Keywords:** ischemic stroke, reperfusion, neuroprotection, nerinetide, Kv2.1

## Abstract

Achieving neuroprotection in ischemic stroke patients has been a multidecade medical challenge. Numerous clinical trials were discontinued in futility and many were terminated in response to deleterious treatment effects. Recently, however, several positive reports have generated the much-needed excitement surrounding stroke therapy. In this review, we describe the clinical studies that significantly expanded the time window of eligibility for patients to receive mechanical endovascular thrombectomy. We further summarize the results available thus far for nerinetide, a promising neuroprotective agent for stroke treatment. Lastly, we reflect upon aspects of these impactful trials in our own studies targeting the Kv2.1-mediated cell death pathway in neurons for neuroprotection. We argue that recent changes in the clinical landscape should be adapted by preclinical research in order to continue progressing toward the development of efficacious neuroprotective therapies for ischemic stroke.

## 1. Introduction

Stroke is a devastating neuropathology associated with immense comorbidity and mortality [1]. It is the second leading cause of death worldwide [1], and more than half of the 18 million people that suffer from a stroke globally each year will have permanent motor deficits reflecting the irreversible loss of neurons [2]. Relative to all neurological disorders, stroke is responsible for the greatest loss of disability-adjusted life years (DALYs) [1]. This represents a tremendous societal burden that is growing constantly with the global population. Despite the broad impact of this pathology, no therapeutic agent has clearly demonstrated the capability to provide neuroprotection and reliably preserve neurological functions in the clinical setting. This gap in medical knowledge highlights the critical need for the development of novel and creative neuroprotective approaches.

Historically, neuroprotective agents are notorious for demonstrating efficacy across several in vitro assays and animal models before failing universally in late-stage clinical trials [3,4]. Many factors have been attributed to this translational failure, including inadequacy of preclinical modeling, inopportune clinical trial design, failure to combine neuroprotectant use with existing reperfusion techniques, and limited knowledge of relevant stroke physiology. However, recent advances in the clinical standard of care for ischemic stroke coupled with advances in the understanding of the relevant pathophysiological mechanisms beckon a new role for effective neurotherapeutic agents. In this review, we describe several critical clinical trials in recent years that have set the landscape for emerging ischemic stroke therapies in the era of late time point reperfusion, and we reflect upon these lessons within the context of our own work in targeting the voltage-gated potassium channel Kv2.1 for neuroprotection. The overarching goal of this review is to capture today’s rapidly evolving clinical landscape in stroke management along with the evolving technology in neuroprotection.

## 2. Physiology of the Ischemic Penumbra and Penumbral Preservation

Ischemic stroke, representing 87% of all stroke cases [2], is classically defined as a cerebrovascular blockage that results in the formation of a central core infarct with a surrounding ischemic penumbra, which is a collaterally perfused at-risk region that can be rescued. The ischemic penumbra manifests radiographically in magnetic resonance (MR) imaging as a region of mismatch between diffusion-weighted (DW) and perfusion-weighted (PW) imaging modalities, identified by the delayed arrival of an injectable tracer agent indicating a perfusion deficit. Similar estimation of core and penumbra can be made with computed tomography perfusion (CTP) imaging [5]. If no intervention is provided, the ischemic penumbra predictably incorporates into the infarct core as necrotic tissue over a period of hours to days [6,7]. The goal for stroke neuroprotection is based on the fundamental concept of penumbral preservation (also known as penumbral freezing), which is the maintenance of cell viability in the penumbra through an intervention in addition to standard treatments with or without reperfusion. Although little can be done to combat the rapid degeneration of the infarct core following severe ischemia, preclinical studies have shown that the collaterally perfused ischemic penumbra can be targeted with neuroprotectants to improve cell survival [8,9]. Even without reperfusion, extensive evidence suggests that the collateral blood flow in the penumbra is essential for tissue survival and may allow the opportunity for a neuroprotective intervention [10,11]. This was most prominently demonstrated in the recent ImpACT-24B trial (NCT00826059), which found improved outcomes in patients with cortical infarcts who received sphenoplatine ganglion stimulation to enhance collateral blood flow [12] and the SETIN trial (NCT01600235), which identified improved outcomes after induced hypertension in patients ineligible for thrombectomy [13]. We propose that these findings together with recent clinical trials with the specific selection criteria of high ischemic penumbra to infarct core ratio for mechanical endovascular thrombectomy provide key clinical evidences of feasibility for penumbral preservation in stroke patients.

Stroke researchers have committed decades to carefully dissecting the cell death pathways underlying the loss of neuronal tissue within the ischemic penumbra. Indeed, many well-defined molecular mechanisms are activated following the initial metabolic failure resulting from the deprivation of oxygen and glucose. These ischemic penumbra mechanisms include the formation of an excitotoxic environment due to the synaptic accumulation of glutamate and calcium dysregulation [14], as well as delayed caspase- and nuclease-dependent apoptosis [15]. Further, late time point reperfusion can increase the risk for reperfusion injury and hemorrhagic transformation. Ischemia-reperfusion injury has been well characterized in a variety of tissues, including the brain, and is associated with the generation of reactive oxygen species (ROS), contributing to the activation of many cell death cascades [16]. For these reasons, there is a clear need to develop neuroprotectants that specifically target cell death mechanisms after ischemic stroke. Despite the difficulties involved in this translational undertaking, the recent advances in stroke treatments, described below, have reinvigorated the race to develop an efficacious treatment for penumbral preservation.

## 3. Recent Advances in Reperfusion Therapy

### 3.1. Canonical Management of Acute Ischemic Stroke

The mainstay intervention method for ischemic stroke patients is the restoration of blood flow by pharmacological or surgical means. The first effective therapy developed was the administration of intravenous recombinant tissue plasminogen activator (rt-PA), alteplase, which received the US Food and Drug Association (FDA) approval in 1996 [17]. rt-PA activates an endogenous fibrinolytic cascade by cleaving plasminogen to its activated form, plasmin, which in turn degrades fibrin and fibrinogen, leading to the dissolution of intravascular clots and the subsequent reperfusion. Although this intervention is effective, its use is limited to early time points. Administration within 3 h of stroke onset is ideal, with diminished efficacy up to 4.5 h poststroke [18,19]. Moreover, treatment with rt-PA does not always lead to successful reperfusion. In fact, recanalization rates range from 10% to 50% depending on anatomic clot localization [20,21,22]. Furthermore, rt-PA has a significant adverse effect profile and an exhaustive list of contraindications that limit its use in patients. Of all patients with ischemic stroke reaching a hospital, it is estimated that thrombolytic drugs are administered in only 2–3% of cases under canonical guidelines [23].

**Figure 1:**
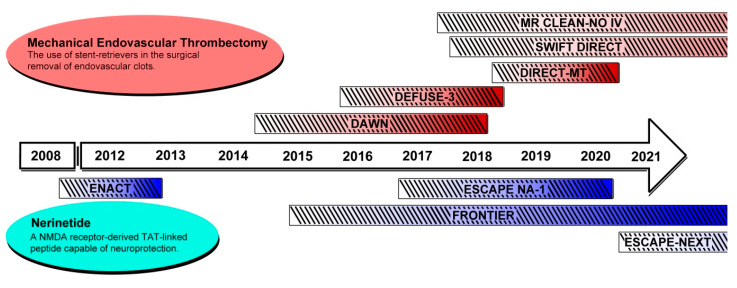
A timeline summary of the clinical trials discussed in this review.

More recently, ischemic stroke management gained the addition of mechanical thrombectomy with endovascular surgeries. This approach involves a surgeon gaining access to the cerebral vasculature, with a stent retriever, an aspiration device, or a combination tool, and physically removing the occlusive thromboembolism [24]. Critically, a series of key studies published in 2015 reported that mechanical endovascular thrombectomy allows superior recanalization rates of up to 88%, with marked improvements in functional outcomes [25,26,27,28,29]. Initial clinical studies focused on validating the efficacy and safety of these surgical methods included time points of up to 6 h [30]. Although this intervention revolutionized stroke management, similar issues reminiscent of pharmacological thrombolytic remained, as only approximately 7% of patients with ischemic stroke reach a medical center in time for surgical revascularization [31,32]. We describe below two recent studies that have drastically improved the use of mechanical thrombectomy by identifying the patient eligibility criteria at significantly delayed time points of up to 24 h from stroke onset [33,34]. Importantly, these studies demonstrated that imaging techniques can be used to identify patients with large proportions of still-viable penumbra. These revascularization methods at later time points set the stage for the development of neuroprotectants that may act synergistically with reperfusion to further extend the intervention window for substantial penumbral preservation. A graphical timeline of important recent and upcoming studies discussed in this review is summarized in Figure 1.

Dates displayed are based on study start date and publication date of the results. Actual study completion date is typically few months prior to publication. More details can be found on clinicaltrials.gov with their respective NCT number. Mechanical endovascular thrombectomy trials (red shaded) are DAWN, NCT02142283; DEFUSE-3, NCT02586415; DIRECT-MT, NCT03469206; SWIFT DIRECT, NCT03192332; and MR CLEAN-NO IV, ISRCTN80619088 (isrctn.com). Nerinetide trials (blue shaded) are ENACT, NCT00728182; ESCAPE-NA-1, NCT02930018; FRONTIER, NCT02315443; and ESCAPE-NEXT, NCT04462536.

### 3.2. DAWN Phase II/III Trial

The efficacy of late time point mechanical thrombolytic reperfusion was evaluated in a landmark clinical trial that took place between 2014 and 2017 (DAWN; NCT02142283). This study selected for patients with high neurological deficits on the NIH Stroke Scale (NIHSS) but relatively small core infarct size at the time from stroke onset of 6–24 h, with the hypothesis that the mismatch between clinical exam and imaging evidence of infarct reflects a high volume of viable penumbra. Three groups of patients were randomized to either mechanical thrombectomy with standard medical therapy (*n* = 107) or standard medical therapy alone (*n* = 99). Standard medical therapy varied by regional and national guidelines [35,36,37,38], but, in general, included evidence-based use of antiplatelet therapy, systemic thrombolysis with intravenous rt-PA when indicated, blood pressure management, and decompressive hemicraniectomy when indicated. All patients had evidence of intracranial ICA or MCA-M1 occlusion on computed tomography (CT) or magnetic resonance (MR) imaging and a mismatch between clinical neurologic deficit and infarct volume identified with imaging, which was adjusted for age (Groups A–C). Group A consisted of patients 80 years or older with NIHSS ≥10 and infarct volume <21 mL, Group B consisted of patients younger than 80 years of age with NIHSS ≥10 with infarct volume <31 mL, and Group C consisted of patients younger than 80 years of age with NIHSS ≥20 and an infarct volume of 31–51 mL. The investigators concluded that endovascular thrombectomy from 6 to 24 h following “last known well” was efficacious and superior when compared to standard medical therapy alone across all subgroups [34]. This was reflected by the primary endpoint showing mean score for disability on the utility-weighted modified Rankin scale at 90 days, which was significantly higher in the thrombectomy-treated group compared to the control group (5.5 thrombectomy plus standard medical therapy vs. 3.4 standard medical therapy alone; adjusted difference by Bayesian analysis, 2.0 points; 95% credible interval, 1.1-3.0; posterior probability of superiority, >0.999), indicating improved functional capacity and decreased prevalence of disability in patients treated with thrombectomy at 6–24 h following “last known well”. Furthermore, the authors reported a significantly higher level of functional independence at 90 days (modified Rankin scale 0–2) with late time point mechanical thrombectomy when compared to standard medical therapy alone (49% thrombectomy plus standard medical therapy vs. 13% standard medical therapy alone; adjusted difference, 33 percentage points; 95% credible interval, 21–44; posterior probability of superiority, >0.999).

### 3.3. DEFUSE-3 Phase III Trial

Soon after the publication of the DAWN trials results, the DEFUSE-3 clinical trial (NCT02586415) provided a second body of evidence for late time point endovascular thrombectomy in ischemic stroke therapy. In contrast to the DAWN trial, the DEFUSE-3 trial focused on an imaging-based approach for the selection of eligible patients. With evidence from prior studies, the DEFUSE-3 investigators hypothesized that patients with high penumbra-core ratios could benefit from thrombectomy and recanalization at late time points following stroke [39,40,41,42]. They utilized the RAPID neuroimaging system composed of CT and MR imaging with perfusion analysis to identify patients with a significant mismatch between infarct core size and ischemic penumbral volume [5].

From 2016 to 2017, the DEFUSE-3 authors used the RAPID neuroimaging platform to identify a total of 182 functionally independent patients with large vessel occlusion (LVO) of the MCA-M1, MCA-M2, or ICA and a viable penumbra that might benefit from delayed reperfusion. Specific neuroimaging criteria included patients with LVO and a main infarct core lesion <70 mL in volume, with mismatch ratio of ischemic tissue to infarct core ≥1.8 and ≥15 mL of mismatched tissue area, representing the ischemic penumbra. Patients were randomized to either standard medical therapy with late endovascular thrombectomy (*n* = 92) or standard medical therapy alone (*n* = 90). Critically, they demonstrated that reperfusion was effective and superior when utilized 6–16 h following symptom onset in this patient subset [33]. Delayed thrombectomy was associated with a favorable shift in the distribution of functional outcomes (unadjusted common OR 2.77) and an increase in the proportion of patients with functional independence at 90 days, defined as modified Rankin scale of 0–2. Patients treated with late endovascular thrombectomy were functionally independent in 45% of cases compared with 17% in the cohort that received standard medical therapy alone. Importantly, no increase in the rate of symptomatic intracranial hemorrhage or serious adverse events was observed with late endovascular thrombectomy.

Together, the DAWN and DEFUSE-3 trials represent critically important steps forward in stroke therapy, as the therapeutic time window for ischemic stroke management has now been significantly extended. The various techniques that identify patients with large penumbra-core volumes mismatch not only have increased the proportion of patients eligible for currently available treatments but also encourage further development of intervention with neuroprotectants that can further provide penumbral preservation. This advancement in our understanding of stroke physiology is beginning to validate the decades of preclinical work on targeting penumbral mechanisms and has reopened the door to properly evaluate neuroprotective agents developed for the purpose of preserving the ischemic penumbra.

## 4. Recent Advances in Stroke Neuroprotective Therapy

### 4.1. Neuroprotective Agents in Stroke

Although hundreds of drugs have been evaluated clinically for effectiveness as a stroke therapy, less than 5% of these molecules have reached the market [43]. Of these, no mechanistically neuroprotective treatment is available in the United States for clinical use. Creative nondrug approaches, such as hypothermia and hyperbaric oxygen therapy, have not been able to demonstrate efficacy in clinical trials, and some trials have been terminated due to increased mortality (see ICTuS 2 hypothermia trial [44]). Despite these daunting odds, many ongoing lines of research remain steadfast on the translation of stroke neuroprotective agents. Recent preclinical work on a PSD-95 protein inhibitor provides evidence that incorporation of the ischemic penumbra into the necrotic core can be halted by targeting excitotoxic mechanisms [45,46]. Although the clinical work involved with this peptide is not yet conclusive [47], it represents the most promising neuroprotectant for stroke patients to date.

### 4.2. PSD-95 Inhibition, Excitotoxicity, and Nerinetide (NoNO Inc.)

The loss of cerebral blood flow during ischemic stroke deprives the brain of glucose and oxygen, collapsing the necessary cellular respiration machineries for adenosine triphosphate (ATP) generation. As a result, many energy-dependent biological functions stall, including the Na^+^/K^+^-ATPase that typically maintains plasma membrane polarization, leading to complete membrane depolarization within minutes [48,49]. In neurons, the depolarized state causes hyperexcitability and the release of excitatory neurotransmitters into the synaptic cleft, which further propagates the depolarization outwards from the infarct core. Overstimulation of Ca^2+^-permeable ion channels leads to the activation of several Ca^2+^-dependent pathways that can be immediately deleterious to neuronal survival.

Neuronal excitotoxicity is primarily mediated by the activation of the Ca^2+^-permeable N-methyl-d-aspartate (NMDA) receptor [50,51]. Downstream from activation, NMDA receptor subunit GluN2B interacts with proteins within the postsynaptic density (PSD) microdomain, including the membrane-associated guanylate-kinase (MAGUK), PSD-95. The PSD-95 PDZ-2 region binds directly to the N-terminal region of neuronal nitric oxide synthase (nNOS), which depends on the binding of the Ca^2+^-activated enzyme calmodulin. The activation of nNOS releases nitric oxide (NO) and the reaction of NO and superoxide (O^−^) anions form the highly toxic peroxynitrite (ONOO^−^). Peroxynitrite has been shown to mediate most of the toxic actions of NO, leading to many mechanisms of cell death, ranging from necrotic to apoptotic [52].

Nerinetide is a neuroprotective agent designed to ameliorate neuronal excitotoxic damage by preventing the activation of nNOS [53]. Previously called TAT-NR2B9c and NA-1, nerinetide is a 31 amino acid peptide-based treatment derived from the isolated C-terminal residues of GluN2B that mediate the essential interaction with PSD-95, conjugated with the cell-permeant transactivator of transcription (TAT) domain from the HIV-1 genome [54]. This design allows nerinetide to be cell- and blood–brain-barrier permeable and competitively binds PSD-95 to disrupt its interaction with GluN2B, thus preventing nNOS activation mediated by the overstimulation of the NMDA receptor [55]. Decades of preclinical studies have validated this neuroprotective strategy before gaining clinical traction [53]. Notably, the first evidence of its neuroprotective action was demonstrated in the standard transient middle cerebral artery occlusion (MCAO) rat stroke model [55]. Further demonstration of the peptide’s action was shown in a nonhuman primate cynomolgus macaques MCAO model [56]. To further evaluate the potential of nerinetide in human patients undergoing endovascular aneurysm repair, a novel model of embolic stroke was developed by the injection of polystyrene spheres in increasing numbers and sizes. Treatment with nerinetide reduced the number and the volume of microstrokes in macaques injected with the polystyrene spheres [57]. Nerinetide has recently shown promise in exploratory analysis of its clinical trial results, providing both hope and important lessons for the development of stroke neuroprotectants.

### 4.3. ENACT Phase II Trial

In a Phase II clinical trial, the safety and efficacy of nerinetide was evaluated in stroke patients undergoing endovascular aneurysm repair (ENACT; NCT00728182) [46]. Between 2008 and 2011, a total of 182 patients were evaluated (*n* = 92 nerinetide; *n* = 93 saline). An initial estimation of ~400 patients necessary to detect significance was reduced by half with the macaque primate data designed to mimic the clinical presentation of microstrokes after aneurysm repair [57]. Patients were given 2.6 mg/kg of nerinetide in 0.9% saline over 10 min immediately after the aneurysm repair. Lesions number and volume were analyzed by diffusion-weighted (DW) and fluid-attenuated inversion recovery (FLAIR) MR imaging. Primary analysis including all patients found that the number of lesions was significantly reduced by nerinetide treatment, but with no changes to lesion volume.

Further examination and stratification of the data based on patient status found an interesting caveat. The effect of nerinetide was most significant in patients with ruptured as opposed to unruptured aneurysms. Considering only patients with ruptured aneurysms, both lesion number and volume was significantly decreased in patients treated with nerinetide. This is not the case when only evaluating patients with unruptured aneurysms, in which case, neither the number nor volume differ between the treatment groups. This physiological efficacy is reflected in the patient behavioral assessment, which shows significantly more nerinetide-treated patients with ruptured aneurysms receiving a minimal NIHSS score of 0–1 (18/18; 100%), compared to saline-treated patients with also ruptured aneurysms (13/19; 68%). On the other hand, the neurological outcome of patients with unruptured aneurysms was virtually identical in both drug and placebo treatment groups.

### 4.4. ESCAPE-NA1 Phase III Trial

In a Phase III clinical trial, nerinetide was evaluated for efficacy in patients experiencing ischemic stroke undergoing rapid endovascular thrombectomy (ESCAPE-NA1; NCT02930018) [45]. This multinational trial took place between 2017 and 2019, with 549 patients receiving nerinetide at the 2.6 mg/kg dose utilized in the ENACT Phase II trial and 556 patients receiving placebo. Nerinetide was administered as soon as possible after randomization (within 60 min from imaging and randomization) and investigators were required to administer the treatment before arterial access closure. Although there was no difference in primary or secondary outcome in the nerinetide- and placebo-treated groups, there was a promising signal of potential efficacy in the subgroup of patients who were not treated with rt-PA.

A larger proportion of nerinetide-treated patients who did not receive rt-PA achieved functional independence with a modified Rankin scale of 0–2 (59.3%; 130/219 vs. 49.8%; 113/227). The infarct volume of patients who did not receive rt-PA was significantly reduced by nerinetide treatment (26.7 mL vs. 39.2 mL), as was mortality (12.8%; 28/219 vs. 20.3%; 46/227). In contrast, patients who received rt-PA in this trial did not exhibit any beneficial responses to nerinetide treatment. In fact, rt-PA drastically reduced plasma concentration of nerinetide, perhaps to subtherapeutic levels. It was reported in the authors’ communications that nerinetide contains amino acid sequences known to be cleaved by plasmin [58] and that this reduction of nerinetide concentration has been observed previously in animals, though they had first hypothesized based on those animal data that nerinetide might still be efficacious after rt-PA [45]. Although much work remains to solidify the role of nerinetide in clinical applications, ENACT and ESCAPE-NA1 provided the most promising evidence yet that neuroprotection in stroke patients is indeed feasible. The definitive efficacy of nerinetide in thrombectomy patients who have not been treated with rt-PA will be tested in the ESCAPE-NEXT trial (NCT04462536), which is targeted to commence in late 2020.

The FRONTEIR Phase III trial (NCT02315443) began in 2015 to evaluate the use of nerinetide in stroke patients within 3 h of stroke, to be administered IV by first responders. The recruitment for this study is still ongoing; the estimated study completion date is mid-2021.

## 5. The Emerging Landscape of Ischemic Stroke Therapy

These above clinical data represent rare occasions of potential success in ischemic stroke therapy. We must consider the evolving clinical context driven by these results and continue the momentum in future research on neuroprotective drugs and stroke therapy. A common theme that connects all the clinical trials presented here is in the careful stratification of the patient population that may have better represented a well-controlled scientific experiment. In the DAWN and DEFUSE-3 trials, advances in imaging techniques allowed the identification of the patient population with large stroke penumbra regions, optimizing the risk-reward of an invasive procedure. This provided a criterion that is far more tangible than the previously, almost subjective, estimation of time from “last known well”. These results drastically expanded the patient population eligible for endovascular reperfusion. Because more stroke patients are eligible to receive endovascular thrombectomy in this “age of reperfusion” [3], preclinical evaluation of drugs in ischemic-reperfusion injury models are becoming increasingly relevant.

This stratification of the patient population in both clinical trials for nerinetide was also the critical factor in unmasking the drug’s effects. The ENACT trial found significant differences in drug effect based on whether the patient undergoing aneurysm repair sustained a ruptured aneurysm. The ESCAPE-NA1 trial found the unexpectedly strong effect of rt-PA reduces blood nerinetide concentrations to below the therapeutic level. These considerations have allowed nerinetide to continue its clinical development, and it has the potential to be the first drug to demonstrate robust neuroprotection—given the optimal conditions. Decades of extensive basic science and highly specific experimental designs in both the preclinical and clinical setting contributed to this potential success and are necessary to continue the momentum. This is clearly demonstrated in the development of the novel emboli stroke macaque model [57], reflecting its specifically paired human clinical trial [46]. Most importantly, the nerinetide trials provided the most enticing evidence that neuroprotection through pharmaceutical targeting is a feasible therapy for ischemic stroke patients.

These impactful clinical trials provide important hints on what must be accomplished in the development of stroke therapy in the near future, especially in the field of neuroprotection. Encouraged by the positive outlook for nerinetide, we have incorporated elements of these studies in our own research, focusing on the translational targeting of the well-studied neuronal cell death pathway modulated by the voltage-gated potassium channel Kv2.1. We highlight our preclinical progress below.

## 6. Targeting Kv2.1 for Neuroprotection

### 6.1. An Omnipresent Cell Death Mechanism in Neurodegeneration

**Figure 2:**
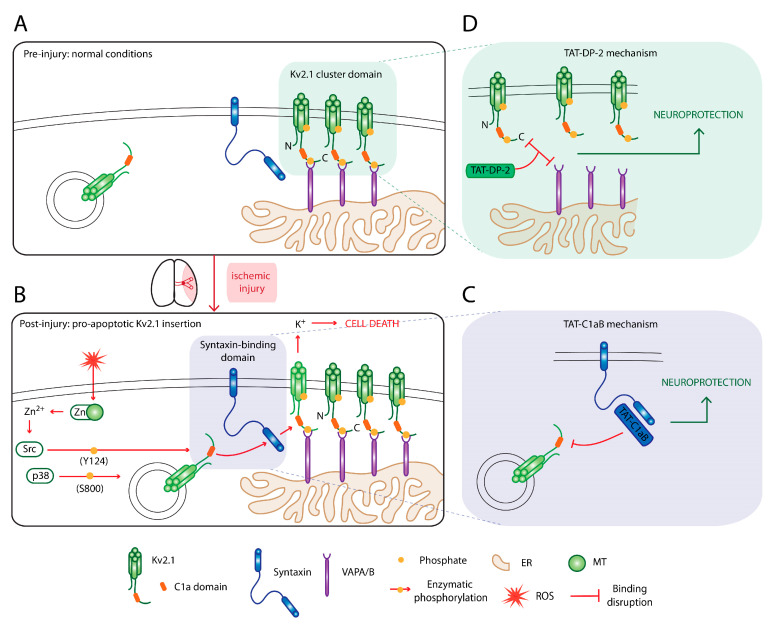
Kv2.1-mediated neuronal cell death and two strategies for neuroprotection. (**A**) In a healthy neuron, Kv2.1 forms somatodendritic clusters with the ER proteins VAPA/B. (**B**) After exposure to proapoptotic stimuli, a zinc-mediated phosphorylation cascade leads to enhanced Kv2.1–syntaxin interaction, thus increasing channel insertion at Kv2.1 channel clusters, enhancing potassium efflux, and enabling cell death mechanisms. This process can be halted to provide neuroprotection in several ways, including (**C**) disrupting Kv2.1–syntaxin binding with TAT-C1aB and (**D**) dispersing Kv2.1 channel cluster with TAT-DP-2 that interferes with Kv2.1-VAPA/B association. MT: metallothionine; ER: endoplasmic reticulum; ROS: reactive oxygen species.

The depletion of intracellular potassium has been shown to be an essential event in the activation of cell death machineries, including APAF-1 apoptosome formation, caspase activation, and nuclease activity [59]. Indeed, changes to potassium efflux has been observed in many preclinical models of neurodegeneration, including stroke [60], traumatic brain injury [61], Parkinson’s disease [62], and Alzheimer’s disease [63,64]. Over the past 20 years, our laboratory has characterized the molecular signaling pathway that is initiated by lethal oxidative damage to deplete intracellular potassium by efflux through the voltage-gated potassium channel Kv2.1. This cell death cascade is initiated by the release of intracellular free zinc from damaged metal-binding proteins [65]. The increase in intracellular zinc activates several phosphorylation pathways that surmise in the phosphorylation of Kv2.1 residues Y124 and S800 by the kinases Src and p38, respectively, and in that preferential order [66,67,68]. These channel modification events increase the interaction between Kv2.1 and syntaxin that is seemingly solely necessary for apoptotic trafficking of the channel [69]. A simplified visual summary of this cell death-enabling pathway is provided in Figure 2A,B. Blocking potassium efflux has long been postulated as a promising neuroprotective approach. However, side effects associated with broad-spectrum potassium channel blockers, such as tetraethylammonium bromide [70], have been a crux in the development of a feasible therapy. As a significant advantage in our strategy, many aspects of the molecular events in the Kv2.1 cell death cascade can be targeted for neuroprotection without affecting Kv2.1 basal currents [69,71,72,73]. We present our two most developed strategies below, as illustrated in Figure 2C,D.

### 6.2. Disrupting the Kv2.1–Syntaxin Interaction

Prior to commitment to apoptosis, Kv2.1 is inserted in the plasma membrane via an enhanced interaction with the SNARE protein syntaxin. The Kv2.1–syntaxin interaction appears to not be necessary for the basal trafficking of the Kv2.1 channel. In cells expressing botulinum toxin that totally abrogates SNARE activity, basal Kv2.1 currents can still be observed while the expression of the enhanced proapoptotic current is abolished [69]. Using a peptide-spot array of small Kv2.1 fragments, we were able to isolate the binding sequence of Kv2.1 to syntaxin, from which we generated a TAT-linked peptide (TAT-C1aB) [74]. We showed that intraperitoneal injections of such TAT-linked peptide was not only able to reach the brain vasculature rapidly but it can also provide neuroprotection in the middle cerebral artery occlusion model of ischemic-reperfusion injury [74]. This is the first in vivo evidence that targeting the Kv2.1–syntaxin interaction can be neuroprotective, as it has been shown many times previously in in vitro models. The mechanism described here is highlighted in Figure 2C. This strategy of displacing a protein–protein interaction using endogenous channel-derived sequences with a TAT-linked peptide mirrors the treatment design in the promising nerinetide story.

Unlike the well-studied interaction between GluN2B and PSD-95, the Kv2.1–syntaxin interaction was not molecularly localized on the target protein. In a bid to further understand the Kv2.1–syntaxin interaction and to extend the effectiveness of our approach, we utilized molecular dynamic simulations to dock C1aB onto syntaxin [71]. We localized the Kv2.1–syntaxin interaction to a highly coordinated binding pocket centered on the syntaxin Ha helix. Leveraging these molecular insights, we were able to screen vast libraries of small molecules and identified candidates that recapitulate the molecular interactions of C1aB and are capable of eliciting neuroprotective actions [71]. We believe that this small molecule approach is the natural progression from designer TAT-peptides, as it allows us to rapidly identify novel treatment candidates with the same mechanism of action. The small molecules libraries may be leveraged to identify drugs that are more efficacious, better tolerated, and possibly resist enzymatic degradation.

### 6.3. Disruption of Kv2.1-ER Cluster Junctions

Recent advances in our understanding of the cellular microdomains that contain Kv2.1 channels have provided valuable insights into their role in neuronal cell death and have highlighted yet another unique protein–protein interaction that may be targeted pharmacologically for neuroprotection. Curiously, a subpopulation of nonconducting Kv2.1 channels localize to micronsized somatodendritic clusters on the cell surface [75,76,77]. These clusters represent ER-PM junctions [78,79] that form as a result of Kv2.1 C-terminal interaction with transmembrane VAMP-associated proteins (VAPA/VAPB) located on the ER [80,81]. These clusters act as trafficking hubs for several proteins, including new Kv2.1 channels reaching the membrane [78], which likely include the proapoptotic population discussed above. Studies based on this work have demonstrated that overexpression of the C-terminus of the cognate channel, Kv2.2, induces dispersal of these channel clusters [82], preventing potassium efflux following oxidative injury, and providing neuroprotection in vitro [83]. In a recent study, we exploited knowledge of this proapoptotic Kv2.1 surface insertion mechanism to validate the targeted-disruption of Kv2.1-VAPA association and cluster dispersal as a neuroprotective strategy [73].

In this work, we similarly identified the critical sequence within the Kv2.2 C-terminus that can disrupt Kv2.1-VAPA association, effectively removing the portal of entry for proapoptotic Kv2.1 channels reaching the membrane. As with the TAT-C1aB peptide, we again created a TAT-linked therapeutic peptide based on this sequence (TAT-DP-2). We showed that this peptide, importantly, induces rapid disruption of Kv2.1 channel clusters in mice in vivo following intraperitoneal injection and demonstrated its neuroprotective efficacy in the context of ischemic stroke (Figure 2D). We showed that when administered by intraperitoneal injection following MCAO with subsequent reperfusion, TAT-DP-2 reduces total infarct volume at 24 h and provides long-term preservation of neurological motor function in mice over a 42-day period [73].

Taken together, the results of these studies provide promising evidence for the specific targeting of proapoptotic potassium efflux as an ischemic stroke therapy. With late time point reperfusion becoming a mainstay of clinical stroke treatment, targeting mechanisms such as these may aid in penumbral preservation, increasing the number of patients eligible for endovascular thrombectomy and improving the already positive outcomes that this therapy provides.

## 7. Other Recent and Ongoing Clinical Trials for Ischemic Stroke Therapy

### 7.1. Mechanical Endovascular Thrombectomy

The optimization of late time point reperfusion in ischemic stroke management is an ongoing process with new clinical trials constantly underway. Recently, the DIRECT-MT trial (NCT03469206) found that endovascular thrombectomy alone was not inferior to combinational treatment with rt-PA and endovascular thrombectomy, although there was a slight improvement with the combined treatment in prethrombectomy reperfusion and overall successful reperfusion [84]. Whether combining rt-PA with thrombectomy is beneficial is being further tested in multiple ongoing trials internationally: see SWIFT DIRECT (NCT03192332) and MR CLEAN-NO IV (ISRCTN80619088). Now more than ever, it is essential for the stroke researcher to be continually monitoring the evolution of clinical techniques in ischemic stroke therapy.

### 7.2. NMDA-Related Neuroprotective Therapies

In the sphere of neuroprotection, NMDA antagonism is one of the most extensively explored strategies, yet many preclinical studies focused on NMDA antagonism have failed to reach clinical translation—often due to their small therapeutic efficacy window, poor safety profile, and failure to target extrasynaptic and prodeath roles of NMDA receptors aside from canonical synaptic ion conduction [85]. In addition to nerinetide, several molecules targeting excitotoxic mechanisms are currently or were recently in the spotlight for clinical evaluations. In a large Phase III clinical trial reported in 2015 (FAST-MAG; NCT00059332), field-administered magnesium sulfate was evaluated as a neuroprotective therapy that acts by blocking NMDA receptors. Despite earlier data suggesting possible efficacy, modified Rankin Score evaluation did not find a favorable shift in neurological deficits from the magnesium sulfate treatment [86], further highlighting the importance of targeting non-conducting NMDA signaling mechanisms. Neu2000 (nelonemdaz), a derivative of sulfasalazine that selectively blocks NMDA and scavenges free radicals, is being evaluated in Phase II clinical trials (SONIC; NCT02831088) [87]. SP-8203, otaplimastat, an earthworm extract protease that appears to elicit neuroprotection through a pleiotropic mechanism that includes blocking NMDA receptors and inhibiting metalloproteinases will be proceeding from Phase IIa to IIb as of 2018 (SAFE-TPA; NCT02787278) [88]. In addition to the nerinetide FRONTIER and ESCAPE-NEXT trials, the SONIC and the SAFE-TPA trials are important upcoming results that will allow us to further elucidate the therapeutic potentials of modulating NMDA receptors in stroke treatment.

Although these ischemic stroke trials primarily focus on direct targeting of synaptic NMDA receptors, newer evidence suggests the importance of targeting more nuanced roles of extrasynaptic NMDA receptors. For instance, activation of extrasynaptic NMDA receptors have been indicated in downregulating the ERK pathway, which ultimately leads to the expression of prodeath genes. A recent review article from Wu and Tymianski in 2018 [85] elegantly suggests that weaker NMDA antagonists may be better suited to selectively target these extrasynaptic channels, providing an adjunct therapy to downregulate proapoptotic gene expression following ischemia. Given the extensive list of preclinical NMDA inhibitors that have failed to reach translation, future focus on this extrasynaptic population of NMDA receptors may provide clarity to the role of NMDA modulation in the context of ischemic stroke therapy.

## 8. Conclusions

The development of an effective and integrated antithrombotic treatment regimen in the clinical treatment of ischemic stroke has been a multidecade effort plagued by challenges and failures, yet highlighted by revolutionary findings that provide meaningful benefits to patients afflicted by this devastating pathology. Nine years after rt-PA was initially approved for thrombolytic use in myocardial infarction, it was finally validated and approved by the US Food and Drug Administration (FDA) for use in ischemic stroke in 1996. After a lull, the advancements in stroke management techniques over the last few years have been accelerating—from the demonstration of the efficacy of endovascular thrombectomy in 2015 to the validation of late time point revascularization up to 24 h poststroke beginning in 2018. Not covered in this review, antiplatelet and antiedema therapies are both massive and immensely promising lines of research. In addition, the diabetes treatments associated with GLP-1 agonism and DPP-4 inhibition have also shown neuroprotective effects in preclinical studies and promising results in reducing major cardiovascular events specifically in type 2 diabetes patients. This literature, including the two recent related clinical trials CARMELINA (NCT01897532) and CAROLINA (NCT01243424), have been reviewed by Darsalia and colleagues [89,90]. In this current translational landscape, the motivation to study and develop novel neuroprotective strategies has been renewed and reinvigorated. With promising neuroprotectant peptides both in preclinical development and displaying possible signs of efficacy in Phase III clinical trials, as is in the case of nerinetide, we may be closer than ever to a novel class of approved and validated neuroprotective agents for ischemic stroke treatment.

## Abbreviations

DALYs	Disability-adjusted life years
MR	Magnetic resonance
DW	Diffusion-weighted
PW	Perfusion-weighted
CT	Computed tomography
ROS	Reactive oxygen species
rt-PA	Recombinate tissue plasminogen activator
NIHSS	NIH stroke scale
ICA	Internal carotid artery
MCA	Middle cerebral artery
LVO	Large vessel occlusion
ATP	Adenosine triphosphate
NMDA	N-methyl-D-aspartate
PSD	Postsynaptic density
MAGUK	Membrane-associated guanylate-kinase
nNOS	Neuronal nitric oxide synthase
NO	Nitric oxide
O^−^	Superoxide
ONOO^−^	Peroxynitrite
TAT	Transactivator of transcription
MCAO	Middle cerebral artery occlusion
FLAIR	Fluid-attenuated inversion recovery MR imaging
MT	Metallothionine
SNARE	SNAP receptor
ER-PM	Endoplasmic reticulum-plasma membrane
VAP	VAMP-associated protein

## References

[1] Johnson C.O., Nguyen M., Roth G.A., Nichols E., Alam T., Abate D., Abd-Allah F., Abdelalim A., Abraha H.N., Abu-Rmeileh N.M. (2019). Global, regional, and national burden of stroke, 1990–2016: A systematic analysis for the Global Burden of Disease Study 2016. Lancet Neurol..

[2] Benjamin E.J., Blaha M.J., Chiuve S.E., Cushman M., Das S.R., Deo R., Floyd J., Fornage M., Gillespie C., Isasi C. (2017). Heart disease and stroke statistics-2017 update: A report from the American Heart Association. Circulation.

[3] Savitz S.I., Baron J.-C., Yenari M.A., Sanossian N., Fisher M. (2017). Reconsidering Neuroprotection in the Reperfusion Era. Stroke.

[4] Savitz S.I., Fisher M. (2007). Future of neuroprotection for acute stroke: In the aftermath of the SAINT trials. Ann. Neurol..

[5] Straka M., Albers G.W., Bammer R. (2010). Real-time diffusion-perfusion mismatch analysis in acute stroke. J. Magn. Reson. Imaging.

[6] Hakim A.M. (1987). The cerebral ischemic penumbra. Can. J. Neurol. Sci..

[7] van der Worp H.B., van Gijn J. (2007). Acute Ischemic Stroke. N. Engl. J. Med..

[8] Bråtane B.T., Cui H., Cook D.J., Bouley J., Tymianski M., Fisher M. (2011). Neuroprotection by freezing ischemic penumbra evolution without cerebral blood flow augmentation with a postsynaptic density-95 protein inhibitor. Stroke.

[9] Baron J.-C. (2018). Protecting the ischaemic penumbra as an adjunct to thrombectomy for acute stroke. Nat. Rev. Neurol..

[10] Wufuer A., Wubuli A., Mijiti P., Zhou J., Tuerxun S., Cai J., Ma J., Zhang X. (2018). Impact of collateral circulation status on favorable outcomes in thrombolysis treatment: A systematic review and meta-analysis. Exp. Ther. Med..

[11] Iwasawa E., Ichijo M., Ishibashi S., Yokota T. (2016). Acute development of collateral circulation and therapeutic prospects in ischemic stroke. Neural Regen. Res..

[12] Bornstein N.M., Saver J.L., Diener H.C., Gorelick P.B., Shuaib A., Solberg Y., Thackeray L., Savic M., Janelidze T., Zarqua N. (2019). An injectable implant to stimulate the sphenopalatine ganglion for treatment of acute ischaemic stroke up to 24 h from onset (ImpACT-24B): An international, randomised, double-blind, sham-controlled, pivotal trial. Lancet Lond. Engl..

[13] Bang O.Y., Chung J.W., Kim S.K., Kim S.J., Lee M.J., Hwang J., Seo W.K., Ha Y.S., Sung S.M., Kim E.G. (2019). Therapeutic-induced hypertension in patients with noncardioembolic acute stroke. Neurology.

[14] Sattler R., Tymianski M. (2001). Molecular mechanisms of glutamate receptor-mediated excitotoxic neuronal cell death. Mol. Neurobiol..

[15] Broughton B.R., Reutens D.C., Sobey C.G. (2009). Apoptotic mechanisms after cerebral ischemia. Stroke.

[16] Nour M., Scalzo F., Liebeskind D.S. (2012). Ischemia-reperfusion injury in stroke. Interv. Neurol..

[17] Disorders N.I.o.N., Group S.r.-P.S.S. (1995). Tissue plasminogen activator for acute ischemic stroke. N. Engl. J. Med..

[18] Powers W.J., Rabinstein A.A., Ackerson T., Adeoye O.M., Bambakidis N.C., Becker K., Biller J., Brown M., Demaerschalk B.M., Hoh B. (2019). Guidelines for the early management of patients with acute ischemic stroke: 2019 update to the 2018 guidelines for the early management of acute ischemic stroke: A guideline for healthcare professionals from the American Heart Association/American Stroke Association. Stroke.

[19] Emberson J., Lees K.R., Lyden P., Blackwell L., Albers G., Bluhmki E., Brott T., Cohen G., Davis S., Donnan G. (2014). Effect of treatment delay, age, and stroke severity on the effects of intravenous thrombolysis with alteplase for acute ischaemic stroke: A meta-analysis of individual patient data from randomised trials. Lancet.

[20] Del Zoppo G.J., Poeck K., Pessin M.S., Wolpert S.M., Furlan A.J., Ferbert A., Alberts M.J., Zivin J.A., Wechsler L., Busse O. (1992). Recombinant tissue plasminogen activator in acute thrombotic and embolic stroke. Ann. Neurol..

[21] Mori E., Yoneda Y., Tabuchi M., Yoshida T., Ohkawa S., Ohsumi Y., Kitano K., Tsutsumi A., Yamadori A. (1992). Intravenous recombinant tissue plasminogen activator in acute carotid artery territory stroke. Neurology.

[22] Menon B.K., Al-Ajlan F.S., Najm M., Puig J., Castellanos M., Dowlatshahi D., Calleja A., Sohn S.-I., Ahn S.H., Poppe A. (2018). Association of clinical, imaging, and thrombus characteristics with recanalization of visible intracranial occlusion in patients with acute ischemic stroke. JAMA.

[23] Adeoye O., Hornung R., Khatri P., Kleindorfer D. (2011). Recombinant tissue-type plasminogen activator use for ischemic stroke in the United States: A doubling of treatment rates over the course of 5 years. Stroke.

[24] Munich S.A., Vakharia K., Levy E.I. (2019). Overview of mechanical thrombectomy techniques. Neurosurgery.

[25] Saver J.L., Goyal M., Bonafe A., Diener H.-C., Levy E.I., Pereira V.M., Albers G.W., Cognard C., Cohen D.J., Hacke W. (2015). Stent-retriever thrombectomy after intravenous t-PA vs. t-PA alone in stroke. N. Engl. J. Med..

[26] Campbell B.C., Mitchell P.J., Kleinig T.J., Dewey H.M., Churilov L., Yassi N., Yan B., Dowling R.J., Parsons M.W., Oxley T.J. (2015). Endovascular therapy for ischemic stroke with perfusion-imaging selection. N. Engl. J. Med..

[27] Goyal M., Demchuk A.M., Menon B.K., Eesa M., Rempel J.L., Thornton J., Roy D., Jovin T.G., Willinsky R.A., Sapkota B.L. (2015). Randomized assessment of rapid endovascular treatment of ischemic stroke. N. Engl. J. Med..

[28] Jovin T.G., Chamorro A., Cobo E., de Miquel M.A., Molina C.A., Rovira A., San Román L., Serena J., Abilleira S., Ribó M. (2015). Thrombectomy within 8 hours after symptom onset in ischemic stroke. N. Engl. J. Med..

[29] Berkhemer O.A., Fransen P.S., Beumer D., Van Den Berg L.A., Lingsma H.F., Yoo A.J., Schonewille W.J., Vos J.A., Nederkoorn P.J., Wermer M.J. (2015). A randomized trial of intraarterial treatment for acute ischemic stroke. N. Engl. J. Med..

[30] Goyal M., Menon B.K., van Zwam W.H., Dippel D.W., Mitchell P.J., Demchuk A.M., Dávalos A., Majoie C.B., van der Lugt A., De Miquel M.A. (2016). Endovascular thrombectomy after large-vessel ischaemic stroke: A meta-analysis of individual patient data from five randomised trials. Lancet.

[31] Chia N.H., Leyden J.M., Newbury J., Jannes J., Kleinig T.J. (2016). Determining the number of ischemic strokes potentially eligible for endovascular thrombectomy: A population-based study. Stroke.

[32] McMeekin P., White P., James M.A., Price C.I., Flynn D., Ford G.A. (2017). Estimating the number of UK stroke patients eligible for endovascular thrombectomy. Eur. Stroke J..

[33] Albers G.W., Marks M.P., Kemp S., Christensen S., Tsai J.P., Ortega-Gutierrez S., McTaggart R.A., Torbey M.T., Kim-Tenser M., Leslie-Mazwi T. (2018). Thrombectomy for stroke at 6 to 16 hours with selection by perfusion imaging. N. Engl. J. Med..

[34] Nogueira R.G., Jadhav A.P., Haussen D.C., Bonafe A., Budzik R.F., Bhuva P., Yavagal D.R., Ribo M., Cognard C., Hanel R.A. (2018). Thrombectomy 6 to 24 hours after stroke with a mismatch between deficit and infarct. N. Engl. J. Med..

[35] Jauch E.C., Saver J.L., Adams Jr H.P., Bruno A., Connors J., Demaerschalk B.M., Khatri P., McMullan Jr P.W., Qureshi A.I., Rosenfield K. (2013). Guidelines for the early management of patients with acute ischemic stroke: A guideline for healthcare professionals from the American Heart Association/American Stroke Association. Stroke.

[36] Committee E.S.O.E., Committee E.W. (2008). Guidelines for management of ischaemic stroke and transient ischaemic attack 2008. Cerebrovasc. Dis..

[37] Hill K. (2008). Australian Clinical Guidelines for Acute Stroke Management 2007: Acute Stroke Guidelines Writing Subgroup on behalf of the National Stroke Foundation Clinical Guidelines for Acute Stroke Management Expert Working Group. Int. J. Stroke.

[38] Casaubon L.K., Boulanger J.-M., Blacquiere D., Boucher S., Brown K., Goddard T., Gordon J., Horton M., Lalonde J., LaRivière C. (2015). Canadian stroke best practice recommendations: Hyperacute stroke care guidelines, update 2015. Int. J. Stroke.

[39] Albers G.W., Goyal M., Jahan R., Bonafe A., Diener H.C., Levy E.I., Pereira V.M., Cognard C., Cohen D.J., Hacke W. (2016). Ischemic core and hypoperfusion volumes predict infarct size in SWIFT PRIME. Ann. Neurol..

[40] Wheeler H.M., Mlynash M., Inoue M., Tipirneni A., Liggins J., Zaharchuk G., Straka M., Kemp S., Bammer R., Lansberg M.G. (2013). Early diffusion-weighted imaging and perfusion-weighted imaging lesion volumes forecast final infarct size in DEFUSE 2. Stroke.

[41] Lansberg M.G., Christensen S., Kemp S., Mlynash M., Mishra N., Federau C., Tsai J.P., Kim S., Nogueria R.G., Jovin T. (2017). Computed tomographic perfusion to predict response to recanalization in ischemic stroke. Ann. Neurol..

[42] Lansberg M.G., Straka M., Kemp S., Mlynash M., Wechsler L.R., Jovin T.G., Wilder M.J., Lutsep H.L., Czartoski T.J., Bernstein R.A. (2012). MRI profile and response to endovascular reperfusion after stroke (DEFUSE 2): A prospective cohort study. Lancet Neurol..

[43] Chen X., Wang K. (2016). The fate of medications evaluated for ischemic stroke pharmacotherapy over the period 1995–2015. Acta Pharm. Sin. B.

[44] Lyden P., Hemmen T., Grotta J., Rapp K., Ernstrom K., Rzesiewicz T., Parker S., Concha M., Hussain S., Agarwal S. (2016). Results of the ICTuS 2 trial (intravascular cooling in the treatment of stroke 2). Stroke.

[45] Hill M.D., Goyal M., Menon B.K., Nogueira R.G., McTaggart R.A., Demchuk A.M., Poppe A.Y., Buck B.H., Field T.S., Dowlatshahi D. (2020). Efficacy and safety of nerinetide for the treatment of acute ischaemic stroke (ESCAPE-NA1): A multicentre, double-blind, randomised controlled trial. Lancet.

[46] Hill M.D., Martin R.H., Mikulis D., Wong J.H., Silver F.L., Milot G., Clark W.M., MacDonald R.L., Kelly M.E., Boulton M. (2012). Safety and efficacy of NA-1 in patients with iatrogenic stroke after endovascular aneurysm repair (ENACT): A phase 2, randomised, double-blind, placebo-controlled trial. Lancet Neurol..

[47] Hankey G.J. (2020). Nerinetide before reperfusion in acute ischaemic stroke: Déjà vu or new insights?. Lancet Lond. Engl..

[48] Tanaka E., Yamamoto S., Kudo Y., Mihara S., Higashi H. (1997). Mechanisms underlying the rapid depolarization produced by deprivation of oxygen and glucose in rat hippocampal CA1 neurons in vitro. J. Neurophysiol..

[49] Siemkowicz E., Hansen A. (1981). Brain extracellular ion composition and EEG activity following 10 minutes ischemia in normo-and hyperglycemic rats. Stroke.

[50] Choi D.W. (1992). Excitotoxic cell death. J. Neurobiol..

[51] Choi D.W., Rothman S.M. (1990). The role of glutamate neurotoxicity in hypoxic-ischemic neuronal death. Annu. Rev. Neurosci..

[52] Ramdial K., Franco M.C., Estevez A.G. (2017). Cellular mechanisms of peroxynitrite-induced neuronal death. Brain Res. Bull..

[53] Ballarin B., Tymianski M. (2018). Discovery and development of NA-1 for the treatment of acute ischemic stroke. Acta Pharmacol. Sin..

[54] Schwarze S.R., Ho A., Vocero-Akbani A., Dowdy S.F. (1999). In vivo protein transduction: Delivery of a biologically active protein into the mouse. Science.

[55] Aarts M., Liu Y., Liu L., Besshoh S., Arundine M., Gurd J.W., Wang Y.-T., Salter M.W., Tymianski M. (2002). Treatment of ischemic brain damage by perturbing NMDA receptor-PSD-95 protein interactions. Science.

[56] Cook D.J., Teves L., Tymianski M. (2012). Treatment of stroke with a PSD-95 inhibitor in the gyrencephalic primate brain. Nature.

[57] Cook D.J., Teves L., Tymianski M. (2012). A translational paradigm for the preclinical evaluation of the stroke neuroprotectant Tat-NR2B9c in gyrencephalic nonhuman primates. Sci. Transl. Med..

[58] Docagne F., Parcq J., Lijnen R., Ali C., Vivien D. (2015). Understanding the functions of endogenous and exogenous tissue-type plasminogen activator during stroke. Stroke.

[59] Shah N.H., Aizenman E. (2014). Voltage-gated potassium channels at the crossroads of neuronal function, ischemic tolerance, and neurodegeneration. Transl. Stroke Res..

[60] Wu K., Yang P., Li S., Liu C., Sun F. (2015). VEGF attenuated increase of outward delayed-rectifier potassium currents in hippocampal neurons induced by focal ischemia via PI3-K pathway. Neuroscience.

[61] Yu W., Parakramaweera R., Teng S., Gowda M., Sharad Y., Thakker-Varia S., Alder J., Sesti F. (2016). Oxidation of KCNB1 potassium channels causes neurotoxicity and cognitive impairment in a mouse model of traumatic brain injury. J. Neurosci..

[62] Redman P.T., Jefferson B.S., Ziegler C.B., Mortensen O.V., Torres G.E., Levitan E.S., Aizenman E. (2006). A vital role for voltage-dependent potassium channels in dopamine transporter-mediated 6-hydroxydopamine neurotoxicity. Neuroscience.

[63] Frazzini V., Guarnieri S., Bomba M., Navarra R., Morabito C., Mariggiò M., Sensi S. (2016). Altered Kv2.1 functioning promotes increased excitability in hippocampal neurons of an Alzheimer’s disease mouse model. Cell Death Dis..

[64] McCord M.C., Aizenman E. (2014). The role of intracellular zinc release in aging, oxidative stress, and Alzheimer’s disease. Front. Aging Neurosci..

[65] Aizenman E., Stout A.K., Hartnett K.A., Dineley K.E., McLaughlin B., Reynolds I.J. (2000). Induction of neuronal apoptosis by thiol oxidation: Putative role of intracellular zinc release. J. Neurochem..

[66] Redman P.T., Hartnett K.A., Aras M.A., Levitan E.S., Aizenman E. (2009). Regulation of apoptotic potassium currents by coordinated zinc-dependent signalling. J. Physiol..

[67] Redman P.T., He K., Hartnett K.A., Jefferson B.S., Hu L., Rosenberg P.A., Levitan E.S., Aizenman E. (2007). Apoptotic surge of potassium currents is mediated by p38 phosphorylation of Kv2.1. Proc. Natl. Acad. Sci. USA.

[68] He K., McCord M.C., Hartnett K.A., Aizenman E. (2015). Regulation of pro-apoptotic phosphorylation of Kv2.1 K+ channels. PLoS ONE.

[69] Pal S., Takimoto K., Aizenman E., Levitan E.S. (2006). Apoptotic surface delivery of K+ channels. Cell Death Differ..

[70] Graham A.J. (1950). Toxic effects of tetraethylammonium bromide. Br. Med. J..

[71] Yeh C.-Y., Ye Z., Moutal A., Gaur S., Henton A.M., Kouvaros S., Saloman J.L., Hartnett-Scott K.A., Tzounopoulos T., Khanna R. (2019). Defining the Kv2.1–syntaxin molecular interaction identifies a first-in-class small molecule neuroprotectant. Proc. Natl. Acad. Sci. USA.

[72] McCord M.C., Kullmann P.H., He K., Hartnett K.A., Horn J.P., Lotan I., Aizenman E. (2014). Syntaxin-binding domain of Kv2.1 is essential for the expression of apoptotic K+ currents. J. Physiol..

[73] Schulien A.J., Yeh C.-Y., Orange B.N., Pav O.J., Hopkins M.P., Moutal A., Khanna R., Sun D., Justice J.A., Aizenman E. (2020). Targeted disruption of Kv2.1-VAPA association provides neuroprotection against ischemic stroke in mice by declustering Kv2.1 channels. Sci. Adv..

[74] Yeh C.-Y., Bulas A.M., Moutal A., Saloman J.L., Hartnett K.A., Anderson C.T., Tzounopoulos T., Sun D., Khanna R., Aizenman E. (2017). Targeting a Potassium Channel/Syntaxin Interaction Ameliorates Cell Death in Ischemic Stroke. J. Neurosci..

[75] O’Connell K.M., Loftus R., Tamkun M.M. (2010). Localization-dependent activity of the Kv2.1 delayed-rectifier K+ channel. Proc. Natl. Acad. Sci. USA.

[76] O’Connell K.M., Rolig A.S., Whitesell J.D., Tamkun M.M. (2006). Kv2.1 potassium channels are retained within dynamic cell surface microdomains that are defined by a perimeter fence. J. Neurosci..

[77] Tamkun M.M., O’Connell K.M., Rolig A.S. (2007). A cytoskeletal-based perimeter fence selectively corrals a sub-population of cell surface Kv2.1 channels. J. Cell Sci..

[78] Deutsch E., Weigel A.V., Akin E.J., Fox P., Hansen G., Haberkorn C.J., Loftus R., Krapf D., Tamkun M.M. (2012). Kv2.1 cell surface clusters are insertion platforms for ion channel delivery to the plasma membrane. Mol. Biol. Cell.

[79] Fox P.D., Haberkorn C.J., Akin E.J., Seel P.J., Krapf D., Tamkun M.M. (2015). Induction of stable ER–plasma-membrane junctions by Kv2.1 potassium channels. J. Cell Sci..

[80] Johnson B., Leek A.N., Solé L., Maverick E.E., Levine T.P., Tamkun M.M. (2018). Kv2 potassium channels form endoplasmic reticulum/plasma membrane junctions via interaction with VAPA and VAPB. Proc. Natl. Acad. Sci. USA.

[81] Kirmiz M., Vierra N.C., Palacio S., Trimmer J.S. (2018). Identification of VAPA and VAPB as Kv2 channel-interacting proteins defining endoplasmic reticulum–plasma membrane junctions in mammalian brain neurons. J. Neurosci..

[82] Baver S.B., O’Connell K.M. (2012). The C-terminus of neuronal Kv2.1 channels is required for channel localization and targeting but not for NMDA-receptor-mediated regulation of channel function. Neuroscience.

[83] Justice J.A., Schulien A.J., He K., Hartnett K.A., Aizenman E., Shah N.H. (2017). Disruption of KV2. 1 somato-dendritic clusters prevents the apoptogenic increase of potassium currents. Neuroscience.

[84] Yang P., Zhang Y., Zhang L., Zhang Y., Treurniet K.M., Chen W., Peng Y., Han H., Wang J., Wang S. (2020). Endovascular Thrombectomy with or without Intravenous Alteplase in Acute Stroke. N. Engl. J. Med..

[85] Wu Q.J., Tymianski M. (2018). Targeting NMDA receptors in stroke: New hope in neuroprotection. Mol. Brain.

[86] Saver J.L., Starkman S., Eckstein M., Stratton S.J., Pratt F.D., Hamilton S., Conwit R., Liebeskind D.S., Sung G., Kramer I. (2015). Prehospital use of magnesium sulfate as neuroprotection in acute stroke. N. Engl. J. Med..

[87] Hong J.M., Choi M.H., Sohn S.-I., Hwang Y.-H., Ahn S.H., Lee Y.-B., Shin D.-I., Chamorro Á., Choi D.W. (2018). Safety and Optimal Neuroprotection of neu2000 in acute Ischemic stroke with reCanalization: Study protocol for a randomized, double-blinded, placebo-controlled, phase-II trial. Trials.

[88] Kim J.S., Lee K.B., Park J.H., Sung S.M., Oh K., Kim E.G., Chang D.i., Hwang Y.H., Lee E.J., Kim W.K. (2019). Safety and Efficacy of Otaplimastat in Patients with Acute Ischemic Stroke Requiring tPA (SAFE-TPA): A Multicenter, Randomized, Double-Blind, Placebo-Controlled Phase 2 Study. Ann. Neurol..

[89] Darsalia V., Klein T., Nyström T., Patrone C. (2018). Glucagon-like receptor 1 agonists and DPP-4 inhibitors: Anti-diabetic drugs with anti-stroke potential. Neuropharmacology.

[90] Darsalia V., Johansen O.E., Lietzau G., Nyström T., Klein T., Patrone C. (2019). Dipeptidyl Peptidase-4 Inhibitors for the Potential Treatment of Brain Disorders; A Mini-Review With Special Focus on Linagliptin and Stroke. Front. Neurol..

